# Supplementary material for: Structural insights into SSNA1 self-assembly and its microtubule binding for centriole maintenance
Source: bioRxiv. 2024 Nov 15:2024.11.13.623454. Preprint. [Version 1] doi: 10.1101/2024.11.13.623454 (PMC11722292; doi:10.1101/2024.11.13.623454)
Supplement: Supplement 3 [file NIHPP2024.11.13.623454v1-supplement-3.pdf]

bioRxiv preprint doi: https://doi.org/10.1101/2024.11.13.623454; this version posted November 15, 2024. The copyright holder for this preprint (which was not certified by peer review) is the author/funder. This article is a US Government work. It is not subject to copyright under 17 USC 105 and is also made available for use under a CC0 license.

Figure S1. Strategy to obtain a structurally amenable form of SSNA-1. A Characterization of SSNA-1(FL-WT). Left: DLS autocorrelation curve with a decay of 5,324.8 μs. Center: Corresponding distribution of hydrodynamic radii of SSNA1(FL-WT). Right: Representative negative-staining EM image of SSNA-1(FL-WT) showing thick filamentous bundles. Scale bar: 100 nm. **B** Characterization of SSNA-1(3E). Left: DLS autocorrelation curve of SSNA-1(3E) in brown with a decay of 15,564.8 μs compared to (FL-WT) from A in blue. Center: Corresponding distribution of hydrodynamic radii of SSNA-1(3E). Right: Representative negative-staining EM image of a thin SSNA-1(3E) filament. Scale bar: 100 nm.

Figure S2. 3D reconstruction of SSNA1(3E). A Cryo-EM data analysis workflow. **B** Local resolution estimation of SSNA-1(3E) with a gold-standard FSC curve for the SSNA-1(3E) cryo-EM reconstruction. **C** Left: 2D class average of SSNA-1(3E) with a box size of 512 pixel (1.648 Å/pixel). Right: Averaged power spectrum of SSNA-1(3E) with layer lines in grey showing the helical periodicity. The measured helical repeat was 112 Å.

Figure S3. Structural analysis and modelling of SSNA1(3E). A Two views of the AlphaFold prediction of SSNA-1(3E) which was fit into the cryo-EM density of a single fibril. Top: The cryo-EM map is colored according to the position of helical strands of the structural model. Bottom: The cryo-EM map is half transparent to show the fitted structural model. Residues M1-S8 are predicted by AlphaFold to be part of the coiled-coil but they would clash when fit into the EM density. The cryo-EM map corresponding to this location shows an extra density from residue F9 protruding to the inner lumen and connecting to the disordered luminal region. **B** Top: AlphaFold prediction of SSNA-1(1-18) folding into the inner lumen of the filament (disordered part in the structure). Bottom: Plot of the predicted alignment error (PAE) of the AlphaFold prediction of SSNA-1(1-18). **C** Side view of the SSNA-1(3E) cryo-EM map where a single fibril connects to the inner lumen density formed by the N-termini. **D** Proportion of non-helical (i.e. disordered) regions as measured by the α-helical contribution in circular dichroism (CD) for SSNA-1(FL-WT) and SSNA-1(12-105). **E** Electrostatic

bioRxiv preprint doi: https://doi.org/10.1101/2024.11.13.623454; this version posted November 15, 2024. The copyright holder for this preprint (which was not certified by peer review) is the author/funder. This article is a US Government work. It is not subject to copyright under 17 USC 105 and is also made available for use under a CC0 license.

surface potential map of the structural model of SSNA-1(3E) residues 7-105 calculated with APBS and PDB2PQR[56] in PYMOL (Schroedinger). The highlighted residues (R18E/R20E/Q98E) were chosen as point mutations to create the structurally amenable form SSNA-1(3E).

Figure S4. Additional biophysical and biochemical characterization of SSNA-1. **A** CD analysis of different SSNA-1 constructs. The average of 4 scans is expressed as ellipticity per mole of peptide residues [θ_R] (deg cm^2 dmol^{-1}). The colors are the same as in panel B. **B** Unfolding curves were measured by CD as a function of temperature with different colors for SSNA1 variants. **C** Schematic of additional SSNA-1 constructs generated to assess functional regions involved in the self-assembly process. **D** Representative negative-staining EM images of SSNA-1(R18E). **E** Representative negative-staining EM image of SSNA-1(Y15E). **F** Representative negative-staining EM image of tubulin (using the conditions of the microtubule-branching assay) showing that microtubules could not be polymerized in the absence of SSNA-1. **G** Representative negative-staining EM image of SSNA-1(11-105). **H** Representative negative-staining EM image of SSNA-1(11-105) mixed with tubulin under microtubule-branching conditions. The yellow asterisk indicates a microtubule branch.

Table S1. Data collection and structure refinement statistics

Table S2. Strains used in this study

Table S3. Repair templates and crRNA sequences used in this study

Movie S1. Time-lapse movie of *ssna-1* WT embryos. The embryos expressed *gfp::histone, mcherry::β-tubulin, and gfp::spd-2* (see also Fig. 1D).

Movie S2. Time-lapse movie of *ssna-1*(Δ) embryos. The embryos expressed *gfp::histone, mcherry::β-tubulin, and gfp::spd-2* (see also Fig. 1D).

bioRxiv preprint doi: https://doi.org/10.1101/2024.11.13.623454; this version posted November 15, 2024. The copyright holder for this preprint (which was not certified by peer review) is the author/funder. This article is a US Government work. It is not subject to copyright under 17 USC 105 and is also made available for use under a CC0 license.

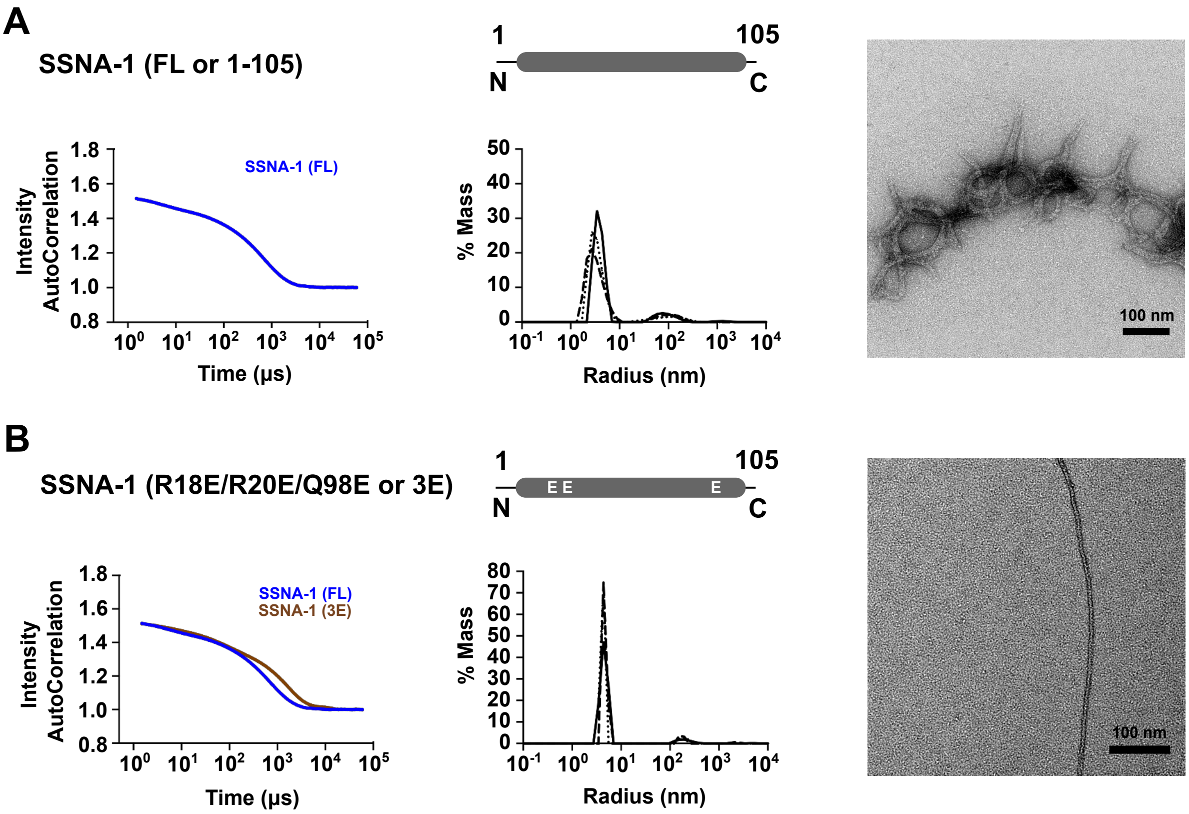

bioRxiv preprint doi: https://doi.org/10.1101/2024.11.13.623454; this version posted November 15, 2024. The copyright holder for this preprint (which was not certified by peer review) is the author/funder. This article is a US Government work. It is not subject to copyright under 17 USC 105 and is also made available for use under a CC0 license.

Figure S2 **Agostini & Pfister et al, 2024**

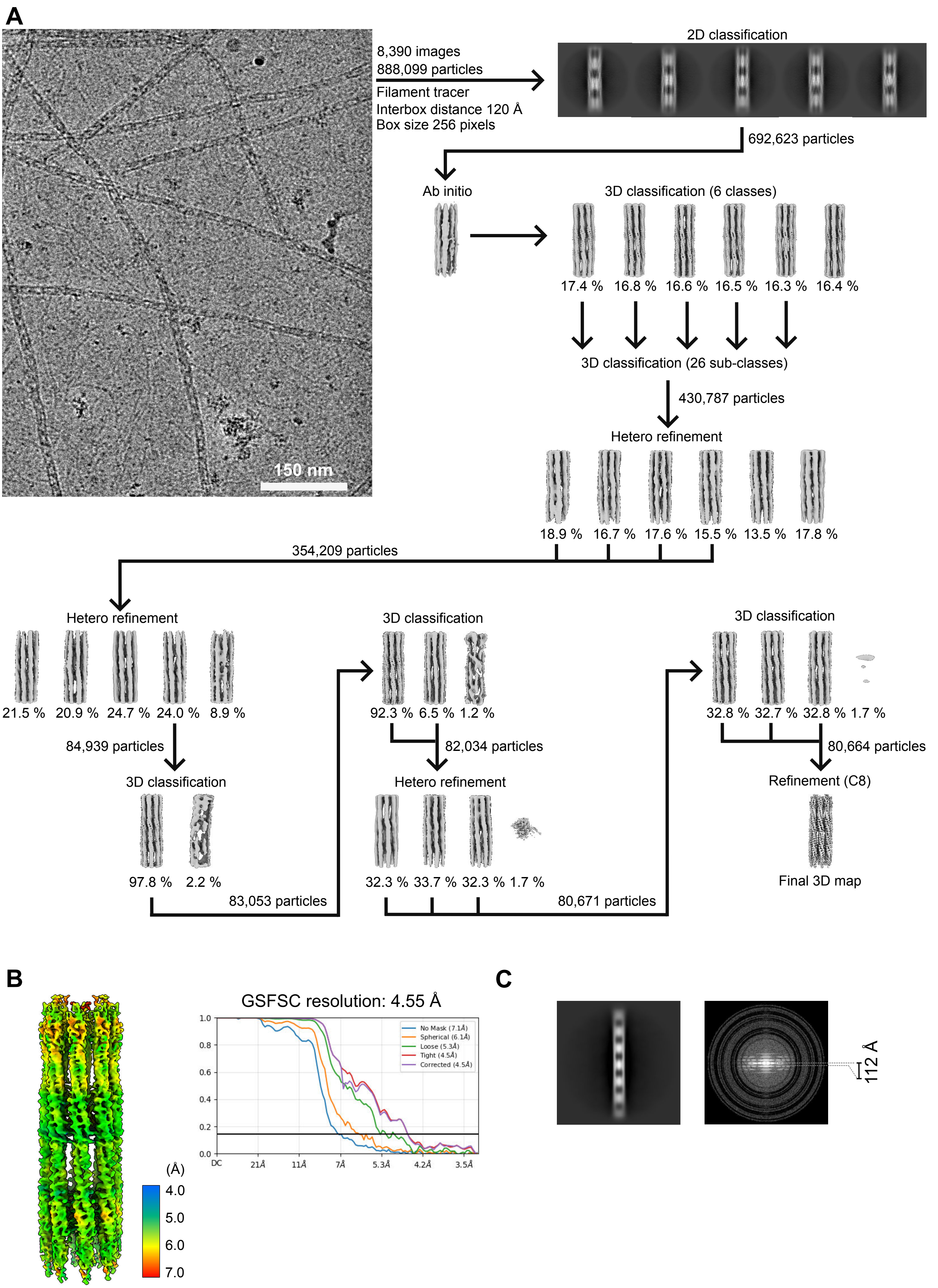

bioRxiv preprint doi: https://doi.org/10.1101/2024.11.13.623454; this version posted November 15, 2024. The copyright holder for this preprint (which was not certified by peer review) is the author/funder. This article is a US Government work. It is not subject to copyright under 17 USC 105 and is also made available for use under a CC0 license.

Figure S3

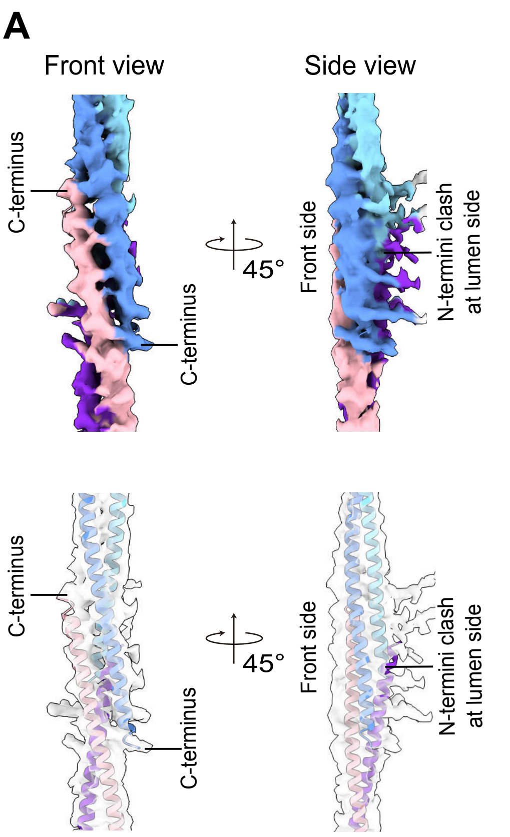

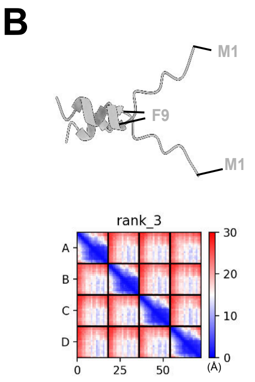

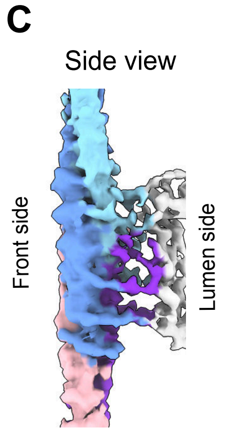

D

Construct	Disordered region (%)
SSNA-1(FL or 1-105)	27.5 %
SSNA-1(12-105)	9.4 %

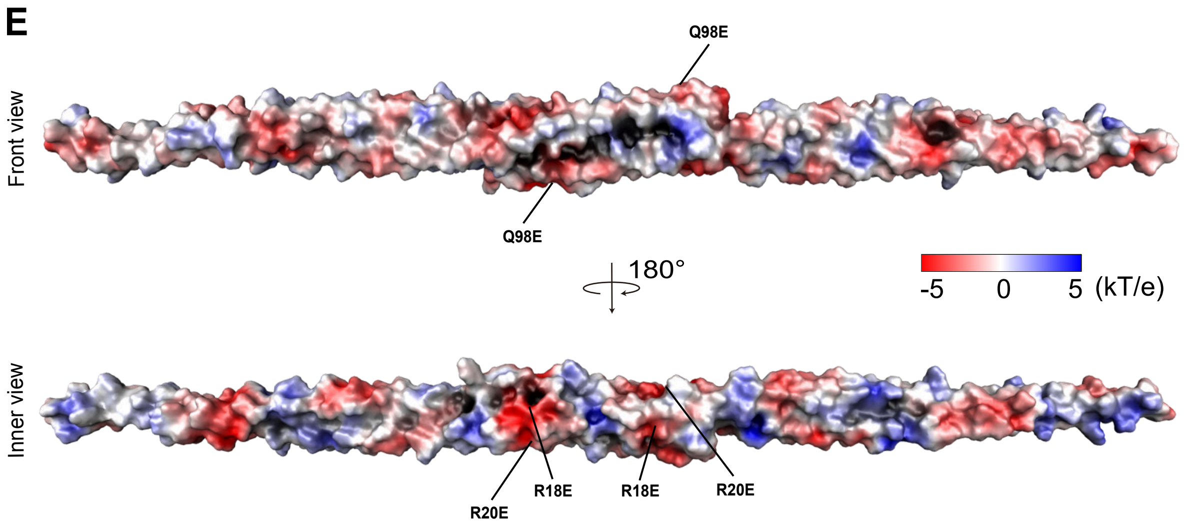

bioRxiv preprint doi: https://doi.org/10.1101/2024.11.13.623454; this version posted November 15, 2024. The copyright holder for this preprint (which was not certified by peer review) is the author/funder. This article is a US Government work. It is not subject to copyright under 17 USC 105 and is also made available for use under a CC0 license.

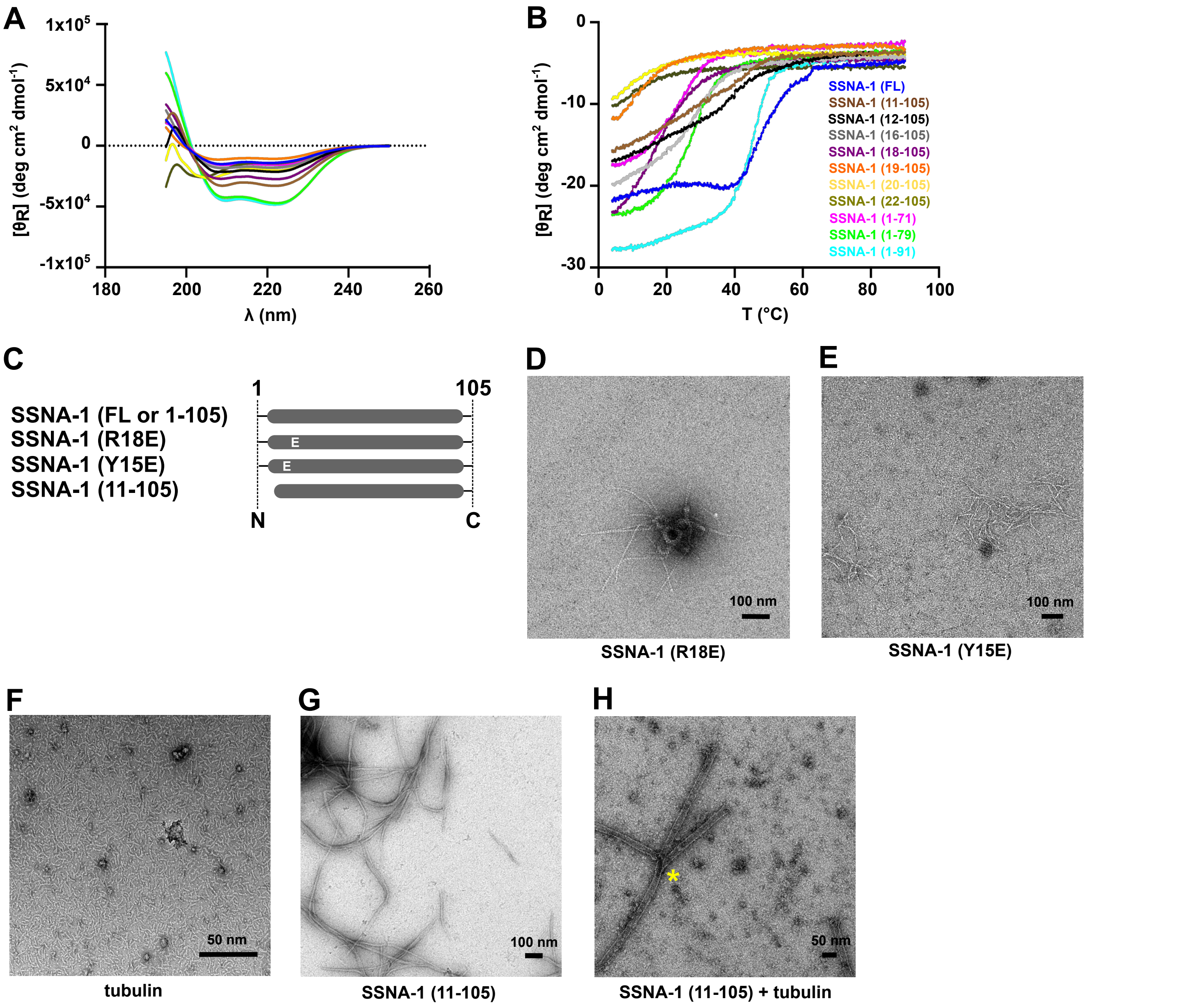

bioRxiv preprint doi: https://doi.org/10.1101/2024.11.13.623454; this version posted November 15, 2024. The copyright holder for this preprint (which was not certified by peer review) is the author/funder. This article is a US Government work. It is not subject to copyright under 17 USC 105 and is also made available for use under a CC0 license.

Table S1: Data collection and structure refinement statistics

**Data Set**	**SSNA-1(R18E/R20E/Q98E)**<br>**EMD-47147**<br>**PDB 9dsm**
**Data collection statistics**	
Microscope	ThermoFisher Scientific Krios G4
Energy filter	Gatan BioContinuum
Detector	Gatan K3 Summit
Grid type	Quantifoil Cu 200 R2/1
Magnification	105,000
Voltage (kV)	300
Electron dose (e^-/Å^2)	40.84
Dose rate (e^-/s/pixel)	14.862
Defocus Range (μm)	-0.5 to -2.3
Number of movie frames	40
Pixel size (Å)	0.412
Number of micrographs	10396
Number of particles (total)	888,099
Number of particles (final map)	80,664
FSC threshold	0.143
Global resolution (Å)	4.55
Local resolution (Å)	3.64 – 36.5
**Refinement statistics**	
Particles used for final map	80,664
Map sharpening B factor (Å^2)	178.83
Number of chains	32
Number of Residues	
Proteins	3168
Number of Atoms	
Proteins	26528
B-factors	
Protein	261.2
R.m.s deviations	
Bond lengths (Å)	0.002
Bond angles (˚)	0.433
Validation	
MolProbity score	1.63
Clashscore	13.27
Rotamer outliers (%)	0.00
CaBLAM outliers (%)	0.00
Cβ outliers (%)	0.00
CC(mask)	0.61
Ramachandran Plot	
Favored (%)	99.7
Allowed (%)	0.3
Outliers (%)	0.0

Table S2. *C. elegans* strains used in this study

N2	Wild type
OC908	*bsSi30[pCW9: unc-119(+) pcdk-11.2::sfgfp::his-58::cdk-11.2 3' utr] II; bsIs20[pNP99: unc-119(+) tbb-1p::mCherry::tbb-2::tbb-2 3'-utr]; bsIs2 [pCK5.5: Ppie-1::gfp::spd-2]*
OC1013	*bsSi30[pCW9: unc-119(+) pcdk-11.2::sfgfp::his-58::cdk-11.2 3' utr] II; bsIs20[pNP99: unc-119(+) tbb-1p::mCherry::tbb-2::tbb-2 3'-utr]; bsIs2 [pCK5.5: Ppie-1::gfp::spd-2]; ssna-1(bs182) / dpy-9(tm9713) kvs-5(tmIs1245)] IV*
OC1021	*zyg-1(bs197[zyg-1::spot] II*
OC1050	*ssna-1(bs218[ssna-1::C-tag]) IV*
OC1051	*zyg-1(bs197[zyg-1::spot] II; ssna-1(bs218[ssna-1::C-tag]) IV*
OC1138	*ssna-1(bs182)/ears-2(ve631[LoxP + myo-2p::GFP::unc-54 3' UTR + rps-27p::neoR::unc-54 3' UTR + LoxP]) IV*
OC1267	*ssna-1(bs284[Y15E]) IV*
OC1274	*ssna-1(bs286[R18E]) IV*
OC1276	*ssna-1(bs312[Y15E, Y97E])/ears-2(ve631[LoxP + myo-2p::GFP::unc-54 3' UTR + rps-27p::neoR::unc-54 3' UTR + LoxP]) IV*
OC1278	*ssna-1(bs314[R18E, Y97E])/ears-2(ve631[LoxP + myo-2p::GFP::unc-54 3' UTR + rps-27p::neoR::unc-54 3' UTR + LoxP]) IV*
OC1336	*ssna-1(bs355[Y97E])/ears-2(ve631[LoxP + myo-2p::GFP::unc-54 3' UTR + rps-27p::neoR::unc-54 3' UTR + LoxP]) IV*

Table S3. Repair templates and crRNA sequences used in this study

<table>
<tr><th>Allele</th><th>Background</th><th>crRNA (5’ -> 3’)</th><th>Repair template (5’ -> 3’)</th></tr>
<tr><td>bs182</td><td>N2</td><td>TAGAATCATGCATTTGCATT
CTTTGTGCGCAAAGAGTATC</td><td>TTCGTATTTGAACAATTACT
GACTAATTTCCTCCGAATG
CAAATGCATGATTCTAGAA
CAAAAAAAACATCAGAAAT
ATTGAACTCTGAACAACTG
TCTC</td></tr>
<tr><td>bs218</td><td>N2</td><td>CTTTGTGCGCAAAGAGTATC
GATATATTTACAGGCATTTT</td><td>GCAAAAGACGTTGGTGGA
CTTTGTGCGCAAAGAGTAT
CAAGATACGAAACATCAGA
AATATGAACCGGAAGCGT
GAACTCTGAACAACTGTCT
CCCAAAAATGCCTGTAAAT
ATATCAATTATCGACATAA
CTTC</td></tr>
<tr><td>bs284</td><td>N2</td><td>AAAATGTCTTCTCGATCTAC
CTGTGAGACGGCGTTCCTCT</td><td>TGCATGATTCTAGAACAAA
AAATGTCTTCTCGAAGCAC
AGGAAGCTTTGATGAAATA
TCACAGGGTAAGAGCAAAT
TGAAGATAAACATTTATAG
TAATATTTCAGAGATCCAA
CGTCTCAGAGAGGAACGC
CGTCTCACAGAATCGTCGA
TTCGAAAAA</td></tr>
<tr><td>bs286</td><td>N2</td><td>CTGTGAGACGGCGTTCCTCT</td><td>GAAGATAAACATTTATAGT
AATATTTCAGACATCCAAG
AGCTCAGAGAGGAACGCC
GTCTCACAGAATCGTCGAT
TCGAAAAATG</td></tr>
<tr><td>bs312</td><td>bs284</td><td rowspan="3">CTTTGTGCGCAAAGAGTATC</td><td rowspan="3">CGCAAAAGACGTTGGTGG
ACTTTGTGCGCAAAGAGGA
GCAGGATACGAAACATCA
GAAATATTGAACTCTGAAC
AAC</td></tr>
<tr><td>bs314</td><td>bs286</td></tr>
<tr><td>bs355</td><td>N2</td></tr>
</table>
